# Importance of Target Gene Locus on the Stability of Recombinant Viruses in the Baculovirus Expression System

**DOI:** 10.3390/v17070902

**Published:** 2025-06-26

**Authors:** Jong Ho Lee, Dong-Hyun Lee, Hyuk-Jin Moon, Soo Dong Woo

**Affiliations:** 1 Department of Agricultural Biology, College of Agriculture, Life & Environment Science, Chungbuk National University, Cheongju 28644, Republic of Korea; willleeam06@naver.com (J.H.L.); 1869oz80@gmail.com (D.-H.L.); qlfmzks@chungbuk.ac.kr (H.-J.M.); 2 IPBL Inc., Cheongju 28644, Republic of Korea; 3 Biomedical Research Institute, Chungbuk National University Hospital, Cheongju 28644, Republic of Korea

**Keywords:** recombinant virus, stability, gene locus, baculovirus, expression system

## Abstract

In the baculovirus expression system, recombinant viruses generated via bacmids often exhibit reduced expression and genetic stability of target genes during serial passages. This instability is thought to arise from the proximity of non-essential exogenous genes to the target gene insertion site. This study investigated the impact of the target gene insertion locus on its expression and stability within the recombinant viral genome. In addition to the conventional *polyhedrin* (*polh*) locus, we evaluated four additional loci located distal to the non-essential exogenous genes. Among them, the *ODV-e56* and *ChiA*/*v-cath* loci maintained target gene expression and genetic stability more effectively than the *polh* and *p10* loci, even after ten consecutive undiluted viral passages. Notably, essential or functionally important viral genes were located near the *ODV-e56* and *ChiA*/*v-cath* loci, whereas such genes were absent near the *p10* locus. These findings suggest that enhanced stability and expression are associated with the proximity to essential viral genes. Therefore, the strategic selection of target gene insertion sites in close proximity to essential viral elements, rather than simply avoiding non-essential exogenous regions, represents a promising strategy to enhance recombinant protein production in baculovirus expression systems.

## 1. Introduction

The baculovirus expression system (BES) utilizes baculoviruses and insect cell lines to produce a wide range of industrially valuable recombinant proteins [1,2,3]. Since the BES relies on eukaryotic insect cells, it enables the production of recombinant proteins with high biological activity through proper post-translational modifications. In the BES, the generation of recombinant viruses carrying the target gene is a key step for producing the desired protein. Recombinant virus generation in the BES can be broadly categorized into two methods based on the recombination mechanism of the target gene [4]. The first is the conventional method, which involves homologous recombination between the viral genome and a transfer vector containing the target gene within insect cells. The second method involves the transposition of the target gene into a bacmid, which is a modified baculovirus genome capable of replicating in *Escherichia coli* due to the incorporation of the mini-F replicon sequence. After selecting recombinant bacmids in *E. coli*, these are transfected into insect cells to generate recombinant viruses.

The homologous recombination method offers the advantage of producing recombinant viruses with high genetic stability of the target gene. However, this approach typically requires labor-intensive and time-consuming virus purification steps within insect cell cultures to ensure the isolation of pure recombinant viruses [4]. In contrast, the bacmid-based transposition method allows for the straightforward selection of pure recombinant bacmids in *E. coli*, thereby simplifying and accelerating recombinant virus production. Nonetheless, recombinant viruses generated by transposition have been reported to exhibit lower genetic stability of the target gene [5,6]. For these reasons, the homologous recombination method is generally preferred for industrial-scale protein production, where stability is critical, while the transposition method is commonly employed for research purposes that demand the rapid generation of multiple recombinant viruses

Recently, efforts have been made to improve the genetic stability of recombinant viruses produced via the transposition method by developing modified bacmids [7]. Previous studies have indicated that the reduced stability of the target gene in transposition-derived recombinant viruses is due to the presence of large exogenous sequences, such as the *E. coli* mini-F replicon and antibiotic resistance genes, which remain in the viral genome [8,9,10]. These bacterial sequences, being non-essential for viral replication or propagation, tend to be naturally deleted during serial virus passaging, often leading to the concurrent loss of adjacent target genes. Based on this understanding, Pijlman et al. [7] developed a novel bacmid in which the target gene was inserted into regions of the viral genome distant from such bacterial sequences. This approach significantly improved the genetic stability of the target gene in the recombinant virus. Specifically, they evaluated four alternative loci for target gene expression: between *v-ubiquitin* and *39K*, between *orf51* and *orf52*, between *gp37* and *DNA polymerase*, and within the *ODV-e56* coding sequence. Among these, only the insertion within the *ODV-e56* locus resulted in a meaningful increase in target gene stability. The authors further suggested that, in addition to spatial separation from bacterial sequences, the enhanced stability might be attributed to the location of the target gene between essential viral genes *IE1* and *IE2*, although no definitive mechanism was proposed.

Therefore, our study aims to further evaluate the importance of the target gene insertion locus in relation to genetic stability in recombinant viruses. Pijlman et al. [7]. had selected target gene loci previously reported to have no adverse effects on gene expression. However, at least two of these loci (between *v-ubiquitin* and *39K*, as well as between *gp37* and *DNA polymerase*) were later found to reduce target gene expression, leading to their exclusion from subsequent studies. Of the four tested loci, three were within untranslated regions (UTRs), and only the insertion within the non-essential *ODV-e56* yielded significant improvements in target gene stability. Given the well-documented functional importance of UTRs, careful evaluation of gene expression levels is essential when selecting these regions for target gene insertion [11,12]. To mitigate the potential risks associated with UTR insertions, our study investigates two alternative loci frequently employed in recombinant protein production to enhance the yield in the BES. The first is the *p10* locus, which is often removed to reduce competition with the target gene promoter or to delay cell lysis, thereby enhancing protein production [13,14,15]. The second target region encompasses the chitinase (ChiA) and cathepsin L-like cysteine protease (v-cath) genes, which are typically deleted to improve recombinant protein stability and yield [16,17]. In addition, this study includes the previously reported loci between *v-ubiquitin* and *39K* (associated with decreased expression) and within *ODV-e56* (associated with increased stability) as reference points for evaluating target gene stability. The impact of these four target gene insertion sites on recombinant protein expression and genetic stability during the serial passaging of recombinant viruses will be systematically assessed.

## 2. Materials and Methods

### 2.1. Cells and Bacmid

*Spodoptera frugiperda* cell lines, Sf9 and transgenic Sf9-QE, were cultured at 27 °C in SFM900II medium (Gibco, Grand Island, NY, USA). For Sf9-QE cells, G418 (Invitrogen, Carlsbad, CA, USA) was supplemented to maintain selection pressure [18]. The bacmid used in this study was bMultibac (Geneva Biotech, Geneva, Switzerland), derived from the *Autographa californica* multiple nucleopolyhedrovirus (AcMNPV-E2; GenBank No. KM667940.1). Routine cell culture maintenance and virus production followed standard protocols [19,20].

### 2.2. λ-Red Recombination

For λ-Red recombination, electrocompetent *E. coli* DH10B_Multibac harboring bMultibac was transformed with pKD46 (GenBank No. AY048746), a plasmid expressing Red recombinase, via electroporation. DNA fragments for recombination were introduced into this *E. coli* following established procedures [21,22,23]. Recombinant bacmids were selected on antibiotic-containing agar plates. To cure pKD46, the selected colonies were incubated at 42 °C for 12 h, and gentamicin-sensitive colonies were isolated. Recombinant bacmids were verified by PCR and DNA sequencing.

### 2.3. Construction of Bacmids Lacking attTn7 Site

To standardize the bacmid structure, the p10 gene and promoter were removed from bMultibac to generate bMultibac-ΔPCC using λ-Red recombination. The upstream and downstream 250 bp regions of the *p10* were PCR-amplified (Table S1) and cloned into pACEBac1 (Geneva Biotech, Geneva, Switzerland) to create pACEBac1-p10 (Figure S1A). An ampicillin resistance gene (Amp^r^), amplified from the pMD20 vector (Takara, Seoul, Republic of Korea), was cloned into pACEBac1-p10 to generate pACEBac1-p10-Amp. DNA fragments amplified from this construct were used for λ-Red recombination. For attTn7 site removal, the lacZ gene containing the attTn7 site was targeted. The flanking 60 bp homologous regions were amplified and used to clone chloramphenicol acetyltransferase (CAT) and sacB genes from pBDC (Addgene #135188, Watertown, MA, USA) to generate recombination fragments (Figure S1B). After recombination, the CAT and sacB genes were removed by introducing DNA fragments containing the upstream 300 bp of CAT and downstream 300 bp of *sacB*, cloned into pMD20-CS (Figure S1C). Recombinants were selected on sucrose-containing antibiotic plates.

### 2.4. Relocation of the attTn7 Site

To reintroduce the attTn7 site at new integration loci, CAT and lacZ genes were amplified from pBDC and bMultibac and cloned into pMD20, resulting in pMD20-CL (Figure S2). The upstream and downstream 250 bp homologous regions of the desired integration sites were PCR-amplified (Table S2) and cloned into pMD20-CL. λ-Red recombination was then performed to relocate attTn7 to the designated positions.

### 2.5. Generation of Recombinant Viruses

The enhanced green fluorescent protein (EGFP) gene was used as the target gene for recombinant virus generation. A previously reported hyper-enhanced expression vector was used to construct a recombinant transfer vector (Figure S3) [24]. This vector was transformed into *E. coli* harboring the bacmid to induce the transposition of the EGFP gene. Recombinant bacmids were selected using blue/white screening based on antibiotic resistance and *lacZ* expression. Recombinant viruses were produced by transfecting Sf9 cells with the recombinant bacmids.

### 2.6. Serial Undiluted Virus Passage

For serial virus passaging, Sf9 cells were seeded at 2 × 10^6^ cells/mL in T25 flasks and infected with 1 mL of passage 1 viral stock. After 3 h of incubation, the inoculum was removed and replaced with 4 mL of fresh medium, followed by a 3-day incubation. The supernatant was collected by centrifugation at 1000× *g* for 5 min. A 1 mL aliquot of the collected supernatant was used to infect fresh cells for subsequent passages. This procedure was repeated for a total of 10 passages.

### 2.7. Fluorescence Intensity Measurement

EGFP-expressing cells infected with recombinant viruses were observed using a fluorescence microscope (Sundew MCXI600 Micros, Veit/Glan, Carinthia, Austria). For quantitative analysis, viral infection was carried out with 1 × 10^6^ cells in 6-well plates at a multiplicity of infection (MOI) of 1. Infected cells were harvested daily, washed with ice-cold PBS, and lysed in 400 μL of lysis buffer (20 mM Tris-HCl, 500 mM NaCl, 1 mM EDTA, 0.1% Tween 20, pH 7.0) supplemented with a protease inhibitor cocktail (Sigma-Aldrich, Burlington, MA, USA) on ice for 30 min. Fluorescence was measured in 96-well plates (100 μL per well) using a Synergy HTX Plate Reader (BioTek Inc., Winooski, VT, USA) with excitation/emission wavelengths of 488/510 nm. Background fluorescence was subtracted using assay buffer alone. All experiments were performed in triplicate.

### 2.8. Virus Titration

Virus titers were determined using the endpoint dilution assay (TCID_50_ method) [19,20]. Sf9 or Sf9-QE cells were seeded in 96-well plates at 1 × 10^4^ cells/well. Serial dilutions of viral supernatants were inoculated into these wells. Infection was assessed at 7 days post-infection through fluorescence microscopy. Virus titers were calculated as plaque-forming units (p.f.u.) according to standard methods [20].

### 2.9. Quantitative PCR (qPCR)

To quantify the EGFP gene copy number, Sf9 cells were infected with recombinant viruses at an MOI of 1. Viral supernatants were harvested on day 7, filtered through a 0.45 μm syringe filter (Corning, Berlin, Germany), and concentrated using a PEG Virus Precipitation Kit (Abcam, Cambridge, UK). Viral pellets were resuspended in PBS and treated with Proteinase K (10 μg/mL) at 45 °C for 1 h. Viral DNA was purified by phenol/chloroform extraction. qPCR was performed using 1 μL of purified viral DNA (15 ng/μL) with primers targeting the EGFP or gp64 gene (Table S1), using the QuantiNova SYBR^®^ Green PCR Kit (QIAGEN, Hilden, North Rhine-Westphalia, Germany) on a Step One Plus Real-Time PCR System (Applied Biosystems, Waltham, MA, USA).

### 2.10. Statistical Analysis

All data were analyzed using SPSS software version 12.0 (SPSS, Inc., Chicago, IL, USA). One-way analysis of variance (ANOVA) followed by the Student–Newman–Keuls (SNK) test was used to assess statistical significance. Data are presented as means ± standard errors (SEs), with significance set at *p* < 0.05.

## 3. Results

### 3.1. Generation of Recombinant Bacmids with Alternative Transposition Sites

**Figure 1:**
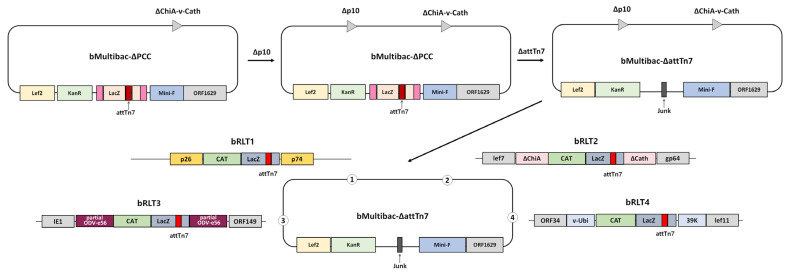
Schematic representation of the recombinant bacmid construction with transposition sites at various loci.

To eliminate factors influencing target gene expression aside from its genomic location, a recombinant bacmid lacking both the *p10* promoter and p10 gene was generated from bMultibac and designated bMultibac-ΔPCC (Figure 1 and Figure S1). Subsequently, the attTn7 transposition site and *LacZ* at the *polh* locus were removed to create bMultibac-ΔattTn7, enabling attTn7 integration at alternative sites (Figure 1 and Figure S1). Using this backbone, recombinant bacmids harboring attTn7 and *LacZ* were constructed at four distinct loci: bRLT1 (*p10* deletion locus), bRLT2 (*ChiA*/*v*-*cath* deletion locus), bRLT3 (*ODV*-*e56* locus), and bRLT4 (between *v-ubiquitin* and *39K*) (Figure 1). These bacmids were used to generate.

**Figure 2:**
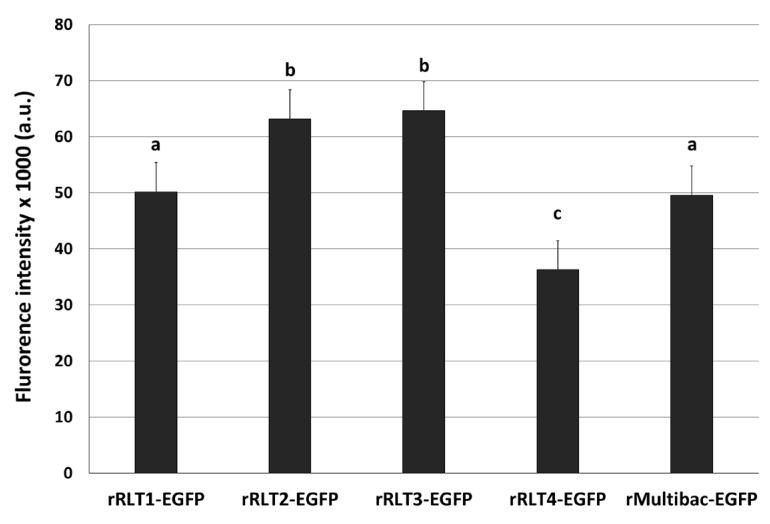
Fluorescence intensity of Sf9 cells infected with recombinant viruses. Recombinant viruses from passage 2, expressing the EGFP, were used. The cells were infected with each virus at an MOI of 1. The fluorescence intensity of the cell extracts was measured using fluorescence photometry at 3 days post-infection and is presented in arbitrary units (a.u.). Values with different letters indicate significant differences (*p* < 0.05; SNK test followed one-way ANOVA).

EGFP-expressing recombinant viruses were named rRLT1-EGFP, rRLT2-EGFP, rRLT3-EGFP, and rRLT4-EGFP, respectively. The control virus derived from bMultibac-ΔPCC was designated rMultibac-EGFP. When EGFP expression was compared, rRLT2-EGFP and rRLT3-EGFP exhibited enhanced expression relative to rMultibac-EGFP, while rRLT1-EGFP showed comparable levels (Figure 2). Conversely, rRLT4-EGFP displayed markedly reduced expression. These results were consistent with previous observations for rRLT3-EGFP and rRLT4-EGFP [7]. Newly evaluated loci, rRLT1-EGFP and rRLT2-EGFP, demonstrated that integration at the *p10* and *ChiA*/*v-cath* loci did not adversely affect expression, with rRLT2-EGFP showing a similar enhancement to that of rRLT3-EGFP. Collectively, these findings highlight that the expression level of a transgene is influenced by its genomic integration site within the baculovirus genome.

### 3.2. Expression Stability of Recombinant Viruses During Serial Undiluted Passages

**Figure 3:**
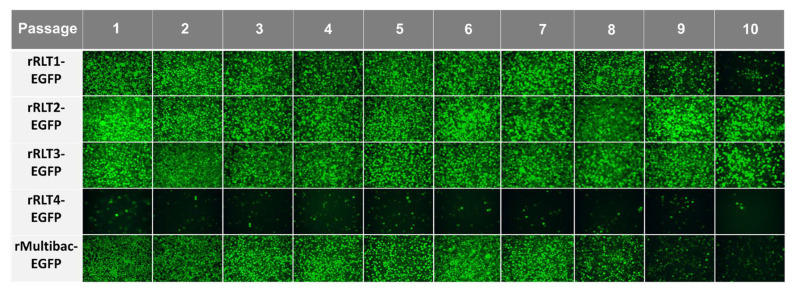
Fluorescence microscopy images of Sf9 cells infected with recombinant viruses at different passages. The cells were observed under a fluorescence microscope at 3 days post-infection following passage without dilution.

To assess expression stability, recombinant viruses were serially passaged up to 10 times without dilution, following a method designed to accelerate instability. Except for rRLT4-EGFP, which showed a consistently low expression, all viruses propagated efficiently, as indicated by the sustained EGFP expression (Figure 3). However, both rMultibac-EGFP and rRLT1-EGFP exhibited a sharp decline in EGFP expression from passage 9, suggesting a progressive loss of recombinant virus stability with serial passaging.

**Figure 4:**
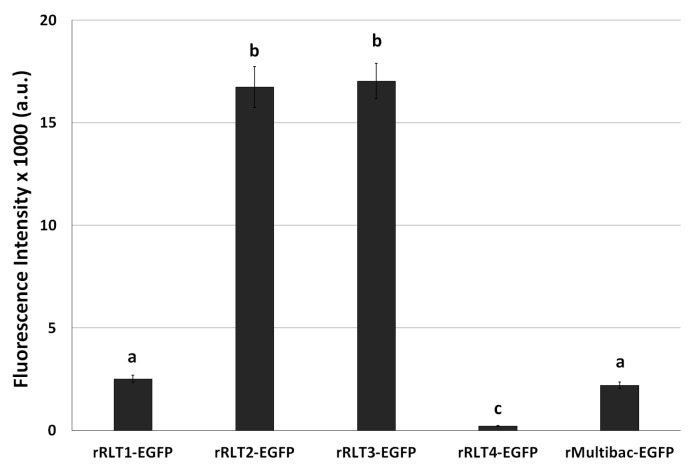
Fluorescence intensity in Sf9 cells infected with recombinant viruses after 10 passages. The cells were infected with each virus at an MOI of 1. The fluorescence intensity of the cell extracts was measured using fluorescence photometry at 3 days post-infection and is shown in arbitrary units (a.u.). Values with different letters are significantly different (*p* < 0.05; SNK test followed one-way ANOVA).

After the 10th passage, EGFP expression levels were again evaluated. rRLT2-EGFP and rRLT3-EGFP retained the highest expression levels, whereas rRLT1-EGFP and rMultibac-EGFP showed similar, lower expression levels (Figure 4). Given its persistently poor expression, rRLT4-EGFP was excluded from further analyses. These results indicate that while rMultibac-EGFP and rRLT1-EGFP exhibited comparable expression stability, rRLT2-EGFP and rRLT3-EGFP maintained significantly greater stability over serial passages. The superior stability of rRLT3-EGFP was in line with previous studies, while the robustness of rRLT2-EGFP was newly demonstrated in this work.

### 3.3. Genetic Stability of Recombinant Viruses After Serial Passaging

**Figure 5:**
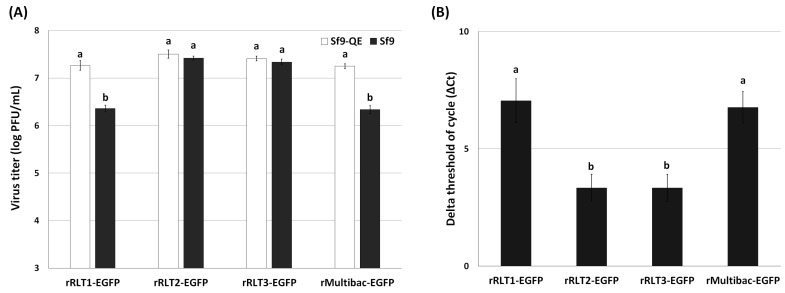
Stability of the EGFP gene in recombinant virus at passage 10. (**A**) The proportion of EGFP-containing recombinant viruses was assessed by comparing viral titers determined by the TCID_50_ method in Sf9-QE and Sf9 cells. (**B**) The relative abundance of the EGFP gene in the viral genomic DNA was quantified by qPCR analysis and the ΔCt method. Values with different letters are significantly different (*p* < 0.05; SNK test followed one-way ANOVA).

To further investigate the genetic stability of these viruses, viral titration assays were conducted after 10 passages using both Sf9 and Sf9-QE cells. Sf9-QE cells, engineered to express the EGFP upon baculovirus infection [18], enable the quantification of both EGFP-positive and EGFP-negative viruses, while Sf9 cells only detect EGFP-expressing viruses. Differences in titers between the two cell lines thus reflect the proportion of EGFP-deleted viruses. The titration results revealed that rMultibac-EGFP and rRLT1-EGFP had over an eightfold higher titer in Sf9-QE cells compared to in Sf9 cells, indicating substantial EGFP deletion events (Figure 5A). In contrast, rRLT2-EGFP and rRLT3-EGFP showed minimal differences (~1.2-fold) between the two cell lines, suggesting high genetic stability. Quantitative PCR analysis further confirmed that rRLT2-EGFP and rRLT3-EGFP maintained higher and comparable EGFP gene copy numbers than rMultibac-EGFP and rRLT1-EGFP (Figure 5B). The relative abundance of the EGFP gene was calculated using the ΔCt method, where ΔCt is defined as the difference between the Ct values of the EGFP gene and the gp64 gene as the reference gene. The reliability of the qPCR was confirmed by melting-curve analysis and the cycle threshold (Ct) values of the EGFP and gp64 genes (Figure S4). Although qPCR analysis indicated the relative abundance of the EGFP gene, it did not allow a direct confirmation of its stability within the viral genome. Therefore, additional analyses of potential genomic deletions, such as PCR or Southern blotting, would provide more definitive evidence regarding the stability of the target gene.

## 4. Discussion

The BES is widely utilized for the production of valuable recombinant proteins in both industrial and research applications. However, a persistent drawback of the BES lies in the limited genetic stability of target genes during serial viral passages [4,5,6,8,9,10]. This issue is particularly pronounced in recombinant viruses generated using bacmids, where the target gene is typically inserted at the *polh* locus. Notably, it has been suggested that the proximity of exogenous sequences adjacent to the integration site exerts a greater influence on viral stability than the choice of the integration site itself [8,9,10]. Recent studies, however, have indicated that altering the integration site of the target gene within the bacmid can enhance the genetic stability of recombinant viruses [7]. In this context, our study evaluated the passage stability of recombinant viruses depending on the target gene’s integration site, aiming to provide practical insights for improving recombinant virus stability through optimized site selection.

A previous report demonstrated that inserting the target gene at a locus distant from non-essential exogenous sequences could enhance viral stability [7]. Building upon these findings, our study assessed not only the previously identified *ODV-e56* locus but also two additional integration sites. Among these, only the *ChiA*/*v-cath* locus conferred a significant increase in the passage stability of the recombinant virus, whereas integration at the *p10* locus failed to enhance stability despite not negatively affecting target gene expression. To understand these observations, we investigated the genetic context surrounding these loci. The *ODV-e56* locus is flanked by *IE1*, an essential gene critical for viral replication [13,14,15,18], and *orf149*, a hypothetical protein of unknown function classified as non-essential [25]. Although prior research has speculated on the role of *IE1* in enhancing viral stability, empirical evidence remains limited to evaluations at this single locus. In the case of the *ChiA*/*v-cath* locus, adjacent genes include *lef7* and *gp64*. While *gp64* is essential for budded virus formation [13,14,15,18], *lef7* is dispensable for viral propagation but crucial for efficient viral DNA replication, with its deletion resulting in reduced replication [26]. Thus, the proximity of such essential or functionally critical genes likely exerts a stabilizing effect on the recombinant virus during serial passages. Importantly, the increased stability of recombinant viruses at these loci is not due to the intrinsic stabilization of the target gene itself. Rather, the concurrent deletion of the target gene along with neighboring essential genes would lead to replication-incompetent viruses, which would be selectively disadvantaged. Consequently, recombinant viruses retaining both essential genes and the target gene would predominate, giving the appearance of enhanced target gene stability. In contrast, the *p10* locus is flanked by *p26* and *p74*, both non-essential genes whose deletions have been associated with increased recombinant protein production [13], suggesting that their loss does not impair viral propagation. As a result, deletion of the target gene at this locus does not confer a selective disadvantage, leading to a decrease in target gene stability over serial passages. Furthermore, unlike previous studies that involved the extensive removal of exogenous sequences, our study retained selectable markers such as *lacZ* and an antibiotic resistance gene in the bacmid to ensure its practical utility. Despite these residual exogenous elements, enhanced stability was only observed when the target gene was integrated near essential viral genes. This underscores the notion that the genetic context, specifically the proximity to essential genes, plays a more decisive role in viral stability than mere spatial separation from non-essential exogenous sequences.

The instability of target genes in recombinant viruses is often attributed to genotypic variations arising from adaptation to artificial cell culture conditions, including the formation of defective interfering particles (DIPs) [9,27]. DIPs, which lack autonomous replication capability, can propagate with the assistance of fully functional viruses. Although the precise mechanisms underlying DIP formation remain unclear, they represent a significant challenge in BES applications, reducing the overall yield of recombinant proteins. Efforts to mitigate DIP formation, such as modifications to the fp25 gene or transposon target sites, have shown partial success, but comprehensive solutions are still lacking [28,29,30]. Therefore, understanding target gene instability requires consideration of both genotypic variations and DIP dynamics.

Nevertheless, our findings suggest that integrating target genes near essential viral genes offers a robust strategy to enhance genetic stability, regardless of these complicating factors. Beyond stability, we also observed that integration at the *ChiA*/*v-cath* locus resulted in increased target gene expression from the early stages of viral propagation. Similar effects were previously reported for the *ODV-e56* locus, where the nearby transcriptional enhancer hr1 was thought to contribute to elevated expression levels [7]. Interestingly, no known enhancers or regulatory elements were identified in the vicinity of *ChiA*/*v-cath*, suggesting alternative, yet-to-be-elucidated mechanisms influencing gene expression at this locus.

Taken together, our results demonstrate that the integration site of a target gene within the bacmid genome can significantly impact both its expression levels and genetic stability. The newly engineered bacmid constructs developed in this study, featuring optimized integration sites, hold promise for enhancing both the yield and stability of recombinant proteins in BES applications. These findings provide valuable insights for the rational design of more stable recombinant baculoviruses for industrial and research purposes.

## Data Availability

The original contributions presented in this study are included in the article/Supplementary Materials; further inquiries can be directed to the corresponding author.

## Supplementary Materials

The following supporting information can be downloaded at https://www.mdpi.com/article/10.3390/v17070902/s1, Figure S1: Schematic representation of recombinant bacmid construction; Figure S2: Schematic representation of the construction of recombinant bacmids with new target gene insertion sites; Figure S3: Structure of a hyper-enhanced expression vector expressing the EGFP; Figure S4: qPCR analysis of the EGFP and gp64 genes in recombinant viruses; Table S1: Primer sequences used in this study; Table S2: Sequences flanking the CAT-LacZ cassette in bRLT.
