# Supplementary material for: Importance of Target Gene Locus on the Stability of Recombinant Viruses in the Baculovirus Expression System
Source: Viruses. 2025 Jun 26;17(7):902. doi: 10.3390/v17070902 (PMC12298668; doi:10.3390/v17070902)
Supplement: Supplementary file 1 [file viruses-17-00902-s001.zip › viruses-3681872-supplementary.pdf]

Table S1. Primer sequences used in this study.

Primer		Nucleotide sequences (5′-3′)
p26	[1]F	GCTGAACGGCGTCCGTGTTG
	[1]R	[3]GACGTCTTAACTATAATATATTGTGTTGGG
p74	F	GGTACCTAAAATTAAACAGCATGCG
	R	AGTTTATGCAGTACACG
Amp	F	GACGTCGACGTCAGGTGGCACTTT
	R	GGTACCGTTACCAATGCTTAATCAG
Cat-SacB	F	CTAAAGCTTAACCAGTGAGCGCAACGCAATTAATGTGAGTTAGCTCA
		CTCATTAGGCACCCCAAGCACACGAGGTCTCTTAGG
	R	ACCAAGCTTCCTGCTTATCCACAACATTTTGCGCACGGTTATGTGGAC
		AAAATACCTGGATGCCAATAGGATATCGGCA
Cat	F	TGATCGGCACGTAAGAGGTTCCAACT
	R	ATTAGATCTTTACGCCCCGCCCTGCCACTCAT
Ac-LacZ	F	AACAGATCTCAGTGAGCGCAACGCAATTAAT
	R	AGATCTCACGGTTATGTGGACAAAATACCTGG
EGFP	F	TCAAGATCCGCCACAACATC
	R	TACTTGTACAGCTCGTCCATGCCGAGAGTGAT
gp64	F	GACTTCGTTCATGTTTGGTCATGTAG
	R	GGTACCTTAATATTGTCTATTACGGTTTCTAAT

[1] Forward primer.

[2] Reverse primer.

[3] Under line indicates restriction endonuclease site.

Table S2. Sequences flanking the CAT-LacZ cassette in bRLT.

Primer		Left flanking sequences (5′-3′)	Right flanking sequences (5′-3′)
RLT1	*p10*	ATGCCGGGACCTTTAATT	ATGAATCGTTTTTAAAATA
RLT2	*ChiA/Cath*	CAACACCGTCCTCCCAC	TCGAAGCCATCATTAAAATG
RLT2	*ODV-e56*	GACGACAAGTGCGCTGCA	ATAACAAGCAGGCCTCGG
RLT4	between *v-ubiquitin* and *39k*	AATAATAAAAACCATTAAAT	ATACATAAAAGTTTTTTAT

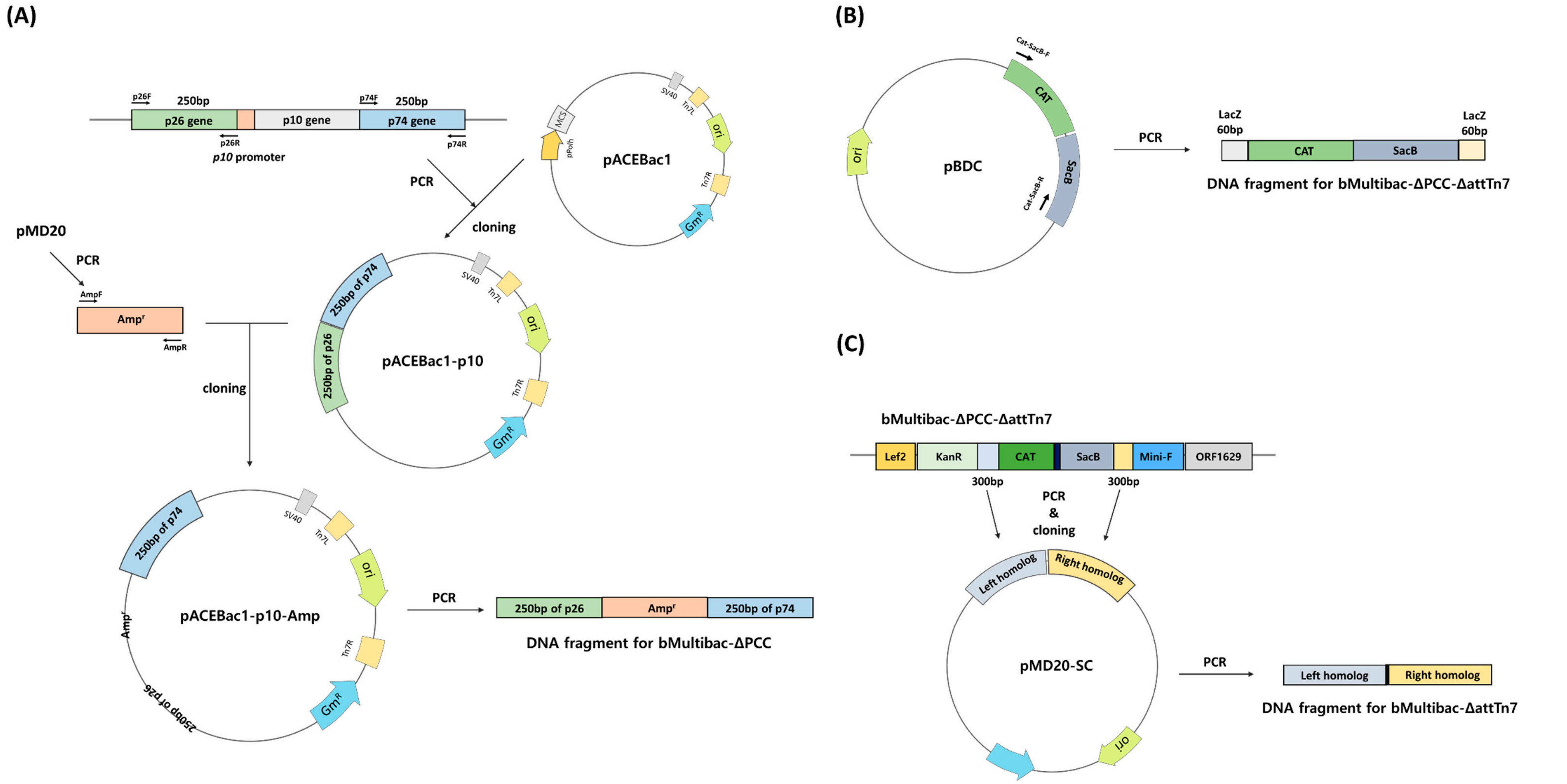

Figure S1. Schematic representation of recombinant bacmid construction. (A) A DNA fragment with a ~250 bp region flanking the *p10* locus and the Amp^r gene was prepared to construct bMultibac-ΔPCC. (B) DNA fragments with 60 bp regions flanking the LacZ gene were prepared to create bMultibac-ΔPCC-ΔattTn7. (C) A DNA fragment was prepared to remove the CAT and SacB genes from the bMultibac-ΔPCC-ΔattTn7 bacmid.

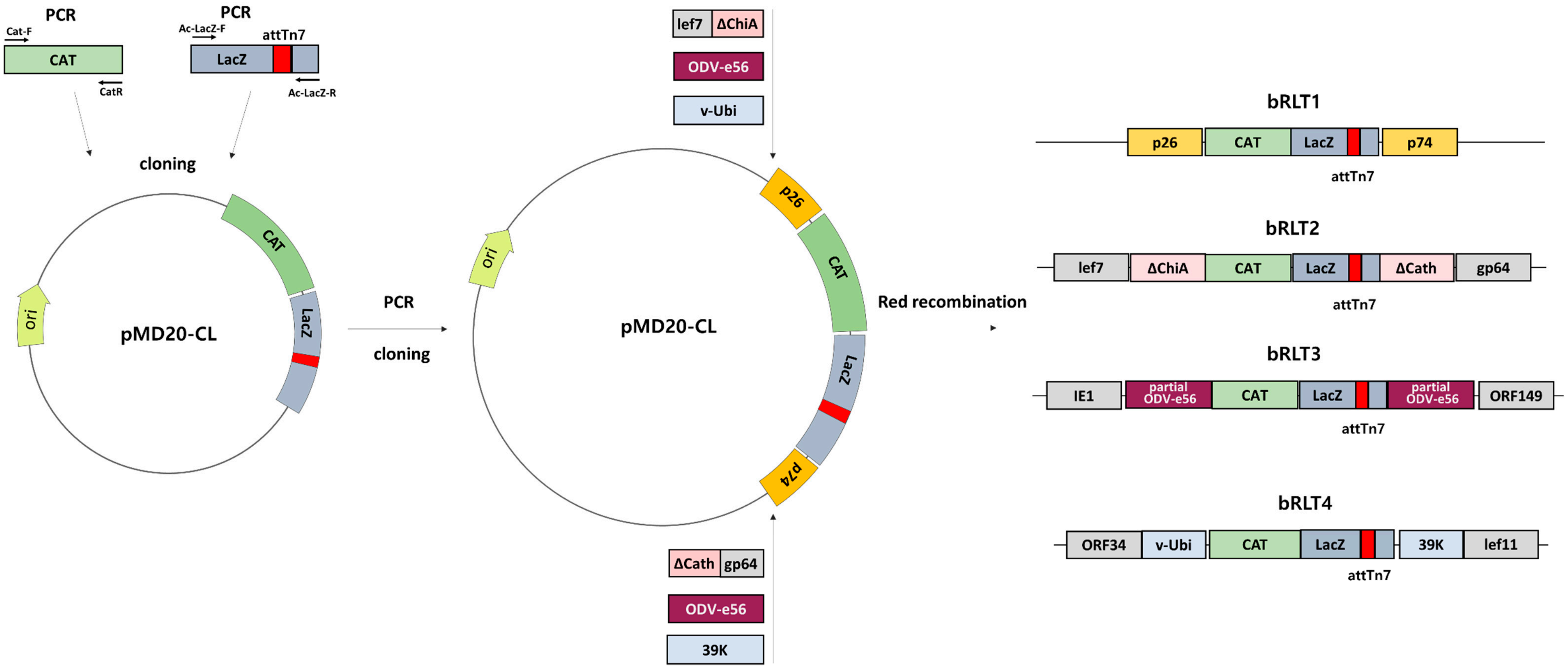

Figure S2. Schematic representation of the construction of recombinant bacmids with new target gene insertion sites. DNA fragments for λ-Red recombination were prepared to insert the attTn7 site at each target expression locus.

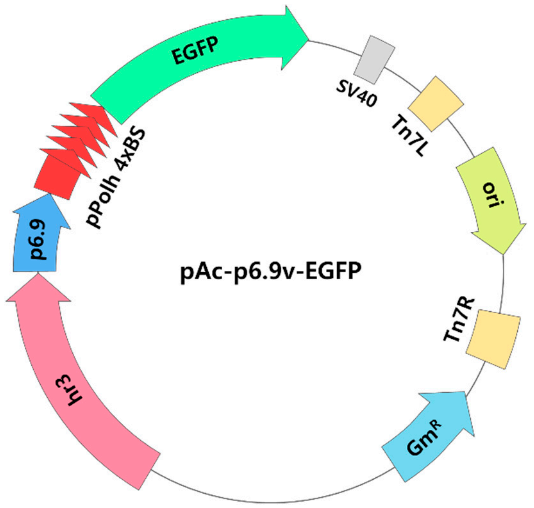

Figure S3. Structure of a hyper-enhanced expression vector expressing EGFP.

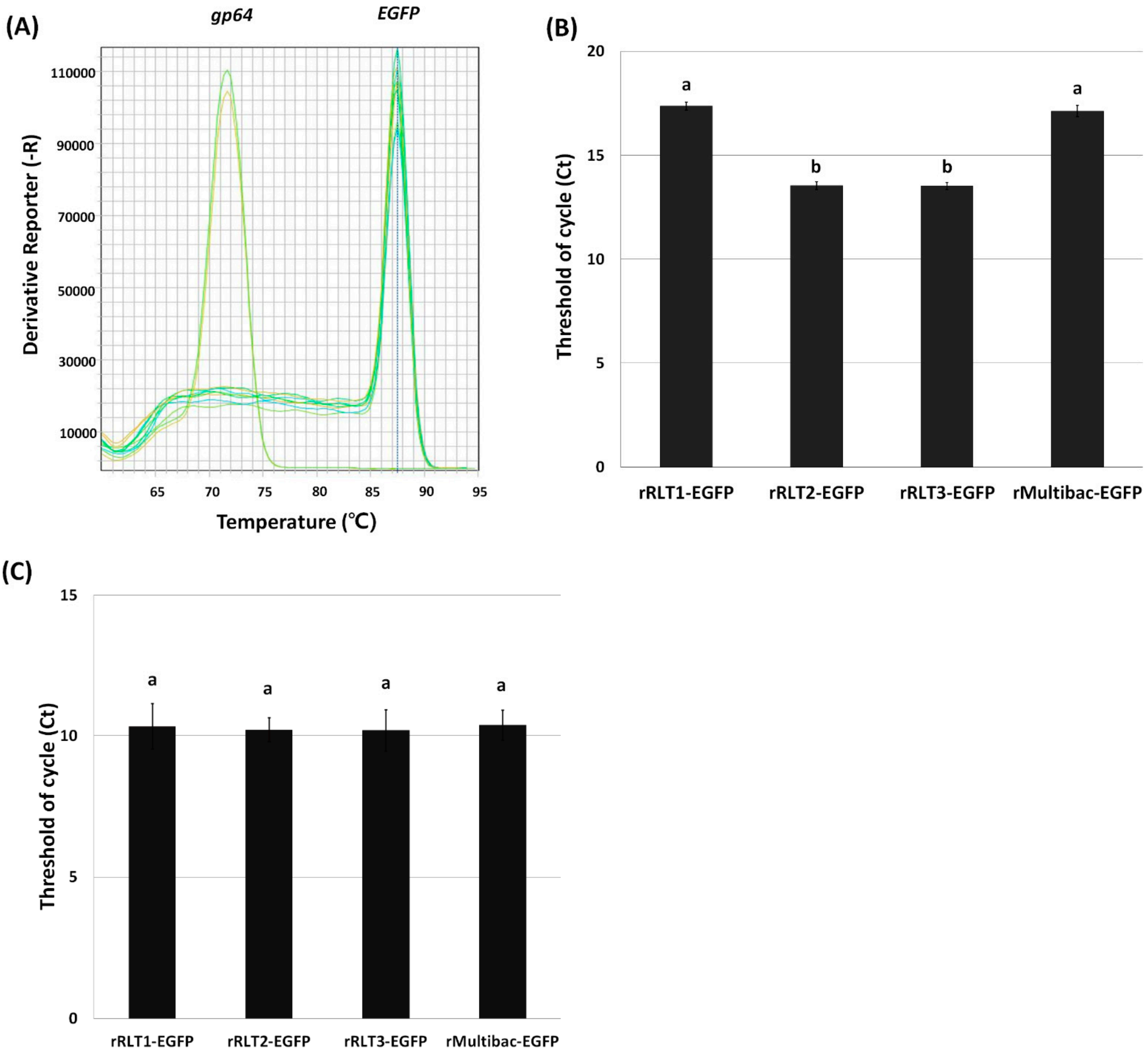

Figure S4. qPCR analysis of the EGFP and gp64 genes in recombinant viruses. To determine the relative copy number of the EGFP gene in recombinant virus genomes, qPCR was performed using gp64 gene as reference gene. The melt curves were analyzed to ensure specific and efficient amplification (A). Cycle threshold (Ct) values for the EGFP (B) and gp64 (C) genes in recombinant viruses were quantified. Values with different letters are significantly differences ($p < 0.05$, SNK test followed one-way ANOVA).
